# Efficacy and safety of abrocitinib for moderate-to-severe atopic dermatitis in adolescents and adults: Meta-analysis

**DOI:** 10.3389/fphar.2023.1154949

**Published:** 2023-05-04

**Authors:** Ling Li, Jiajun Yu, Baoqing Chen, Ying Guo, Yufeng Yang

**Affiliations:** ^1^ Shenzhen Traditional Chinese Medicine Hospital, Shenzhen, China; ^2^ The Eighth Affiliated Hospital of Sun Yat-Sen University, Shenzhen, China

**Keywords:** abrocitinib, atopic dermatitis, dupilumab, meta-analysis, adolescents’

## Abstract

**Objective:** This study aims to investigate the safety and efficacy of abrocitinib in treating moderate-to-severe AD in adolescents and adults.

**Methods:** Pubmed, Cochrane, Embase, and Web of science data base were searched from inception to 9 August 2022. All randomized controlled trials (RCTs) evaluating the efficacy and safety of abrocitinib in moderate to severe AD were included in the meta-analysis.

**Results:** This meta-analysis comprised 7 studies and found that 100 mg or 200 mg of abrocitinib significantly improved IGA {[RR = 2.44, 95% CI (1.93–3.08)] [RR = 3.16, 95% CI (2.52–3.96)]} and EASI-75{[RR = 2.18, 95%CI (1.78–2.67)] [RR = 3.04, 95%CI (2.22–4.16)]} responses compared to placebo. Following that, the population was divided into adolescent and adult groups. The abrocitinib improved IGA, EASI-75 responses, and it was still superior to placebo in both the adolescent and the adult groups. PP-NRS4 response index demonstrated that abrocitinib had a greater effect than placebo at 100 mg [RR = 2.22, 95% CI 1.80–2.72] and 200 mg [RR = 3.28, 95% CI 2.59–4.17]. Abrocitinib improved PSAAD, POEM, DLQI, CDLQI, and HADS more than a placebo.

**Conclusion:** In conclusion, this meta-analysis preliminarily demonstrated that abrocitinib had higher efficacy and safety in the treatment of moderate-to-severe AD in adolescents and adults. In addition, abrocitinib could rapidly relieve itching, and effectively improve symptoms and signs, with a greater effect at the dosage of 200 mg than 100 mg.

## 1 Introduction

Atopic dermatitis (AD) is a chronic, recurrent, inflammatory skin disease. Because patients often have allergic rhinitis, asthma and other atopic diseases, it is considered a systemic disease. AD patients are often characterized by dry skin, eczema-like lesions, severe itching, itchy erythema, and epidermal blisters. AD patients often have severe itching, which seriously affects the quality of life. In recent decades, the prevalence and incidence rate of atopic dermatitis have shown a significant increase (Ständer, 2021), ranging from 2.7% to 20.1%. Among all AD patients, the number of severe AD patients does not exceed 15%, and the prevalence of AD in urban areas is generally higher than that in rural areas (Silverberg et al., 2021). AD ranks 15th among all non-fatal diseases and has the highest disease burden among skin diseases as measured by disability-adjusted life-years (Laughter et al., 2021). Due to its recurrent attacks, skin lesions with exudative tendency, intense itching and other characteristics, and often accompanied by allergic rhinitis, allergic conjunctivitis and asthma, patients can have poor sleep quality, sleep disorders, and even anxiety and depression (Silverberg et al., 2019), which seriously affects patients’ quality of life and physical and mental health (Ständer et al., 2022).

According to the guidelines (Eichenfield et al., 2017; Boguniewicz et al., 2018), it is recommended to use emollients, external corticosteroids, external calcineurin inhibitors and phototherapy for the routine treatment of AD. However, the treatment regimens recommended by the above guidelines usually have no effect on moderate-to-severe patients, and systematic treatment such as immunosuppressive agents (cyclosporine, methotrexate) should be applied when necessary. However, due to the many adverse reactions and poor patient compliance, these treatments cannot be widely promoted and applied. Systemic immunosuppressants are usually not recommended for adolescent AD patients by the current guidelines due to their toxicities (Chu, 2021). In the past few years, two novel systemic therapies have been applied to the treatment of AD: one is antibody against the type 2 inflammation pathway, and the other is the small molecules that inhibit the type 2 pathway and other cytokine signaling. Dupilumab, a subcutaneously administered anti-interleukin-4-receptor α monoclonal antibody, has been approved for the treatment of atopic dermatitis and must be administered parenteral. Abrocitinib is a small-molecule inhibitor of JAK1, which can inhibit the signal transduction, and the current usage is once a day oral administration. Although, a few studies have shown that abrocitinib has good efficacy in the treatment of adolescents and adults with moderate-to-severe atopic dermatitis. But those studies have found variable efficacy and safety among different populations at different doses, and a comparison with Dupilumab was lacking. Based on this situation, we aimed in this meta-analysis to aggregate and quantify the overall efficacy and safety of the drug in adolescents and adults with moderate-to-severe AD.

## 2 Data and methods

This systematic review and meta-analysis follow Cochrane Handbook for the Systematic Review of Interventions (for details, see at http://training.cochrane.org/handbook) and the Preferred Reporting Items for Systematic Review and Meta-analyses (PRISMA) 2020 statement (PMID:1962255215) and pre-registered the research protocol on PROSPERO (CRD42022365878).

### 2.1 Criteria for inclusion and exclusion of literature

#### 2.1.1 Research type

Randomized clinical trials were selected.

#### 2.1.2 Inclusion criteria

① Adolescents and adults AD patients treated with abrocitinib who cannot be adequately controlled by topical or/and systemic therapy during the first 6 months of treatment, or who are not advised to use topical therapy. ② Age ≥12 years, disease course > 1 year, clinical diagnosis of moderate-to-severe AD, [IGA] score ≥3, eczema area and severity index [EASI] score ≥16, affected body surface area ≥10%, and Peak Pruritus Numerical Rating Scale [PP-NRS] score ≥4. ③ Patient’s race, nationality and gender are not limited. The disease diagnosis of AD conforms to Hannifin-Rajka standard.

#### 2.1.3 Exclusion criteria

① Literature not in English; ② Abstract or full text literature cannot be obtained; ③ Original research data cannot be extracted; ④ Inconsistent (missing) studies of interventions or controls; ⑤ Review, abstract, case report and guideline, *etc.*

#### 2.1.4 Intervention measures

According to the randomized controlled double-blind method, patients were divided into: ① Experimental group: oral administration of abrocitinib 100 mg or 200 mg; ② Control group: oral administration of placebo with the same course and method as experimental group or subcutaneous injection of Dupilumab. Other intervention measures were consistent between the experimental group and the control group.

#### 2.1.5 Outcomes

The objective of this meta-analysis is to assess the efficacy and safety of Abrocitinib for moderate-to-severe atopic dermatitis according to the outcomes of the studies. The primary outcomes include: ① IGA (Investigator’s Global Assessment score); ② EASI (Eczema Area and Severity Index); ③ PP-NRS (Peak Pruritus Numerical Rating Scale). The secondary outcomes include: ①POEM(Patient-Oriented Eczema Measure); ②PSAAD (Pruritus and Symptoms Assessment for Atopic Dermatitis); ③DLQI (Dermatology Life Quality Index); ④CDLQI (Children’s Dermatology Life Quality Index); ⑤HADS (Hospital Anxiety and Depression Scale).

### 2.2 Methods

#### 2.2.1 The RCTs about the efficacy and safety of abrocitinib

In the treatment of AD in PubMed, The Cochrane Library, Web of Science and Embase were retrieved by computer from the establishment of the databases to 9 August 2022. The retrieval method took the form of a combination of subject words and free words, and the search keywords were: Abrocitinib and Atopic Dermatitis.

#### 2.2.2 Literature screening and data extraction

Two researchers searched the literature strictly according to the inclusion and exclusion criteria, and then managed all the literature with the software Endnote X9. The retrieved literature was imported into Endnote X9. After the repeated publications were excluded, the preliminary studies were selected by title or abstract, and the full text was downloaded. After reading the full text, the original studies that fit this systematic review were screened. Literature information was extracted and cross-checked to unify the unit of measurement. If there was a dispute, the third researcher was requested to assist in the determination. The contents of data extraction include: ① basic information of the included study: research topic, first author, publication years, *etc.*; ② Baseline characteristics and interventions of subjects; ③ Key elements of bias risk assessment; ④ Outcome indicators and outcome measures of concern.

#### 2.2.3 Risk assessment of bias in included studies

The included studies were independently evaluated for quality by two investigators according to Cochrane Handbook of Systematic Reviews 5.1.0 and the results were cross-checked. The evaluation included randomization methods, assignment concealment, subject and rater blindness, outcome measure blindness, outcome integrity, selection reporting, and other biases. For each assessment, there are three levels of judgment: high (high risk of bias), low (low risk of bias), and unclear (unknown risk of bias).

### 2.3 Statistical methods

Stata 15.0 software was used for statistical analysis of the included literature, including heterogeneity test, publication bias analysis, sensitivity analysis, *etc.* The data extracted in the study were continuous and dichotomous variables with uniform measurement units. The effects were combined with mean difference (MD) and relative risk ratio (RR), and 95% confidence interval (CI) was calculated. Adopted I^2^ Test to evaluate the heterogeneity, if *p* > 0.1 and I^2^ ≤ 50%, indicating that the heterogeneity between studies is acceptable, and the fixed effect model is selected for meta-analysis; If *p* ≤ 0.1 or I^2^> 50%, indicating large heterogeneity between studies, then further analyze the source of heterogeneity. After excluding the influence of obvious clinical heterogeneity, select random effect model for meta-analysis. The “metabias” command was used to test the publication bias of the included studies, and all the results were statistically significant (*p* < 0.05).

## 3 Results

### 3.1 Document screening process and results

**FIGURE 1:**
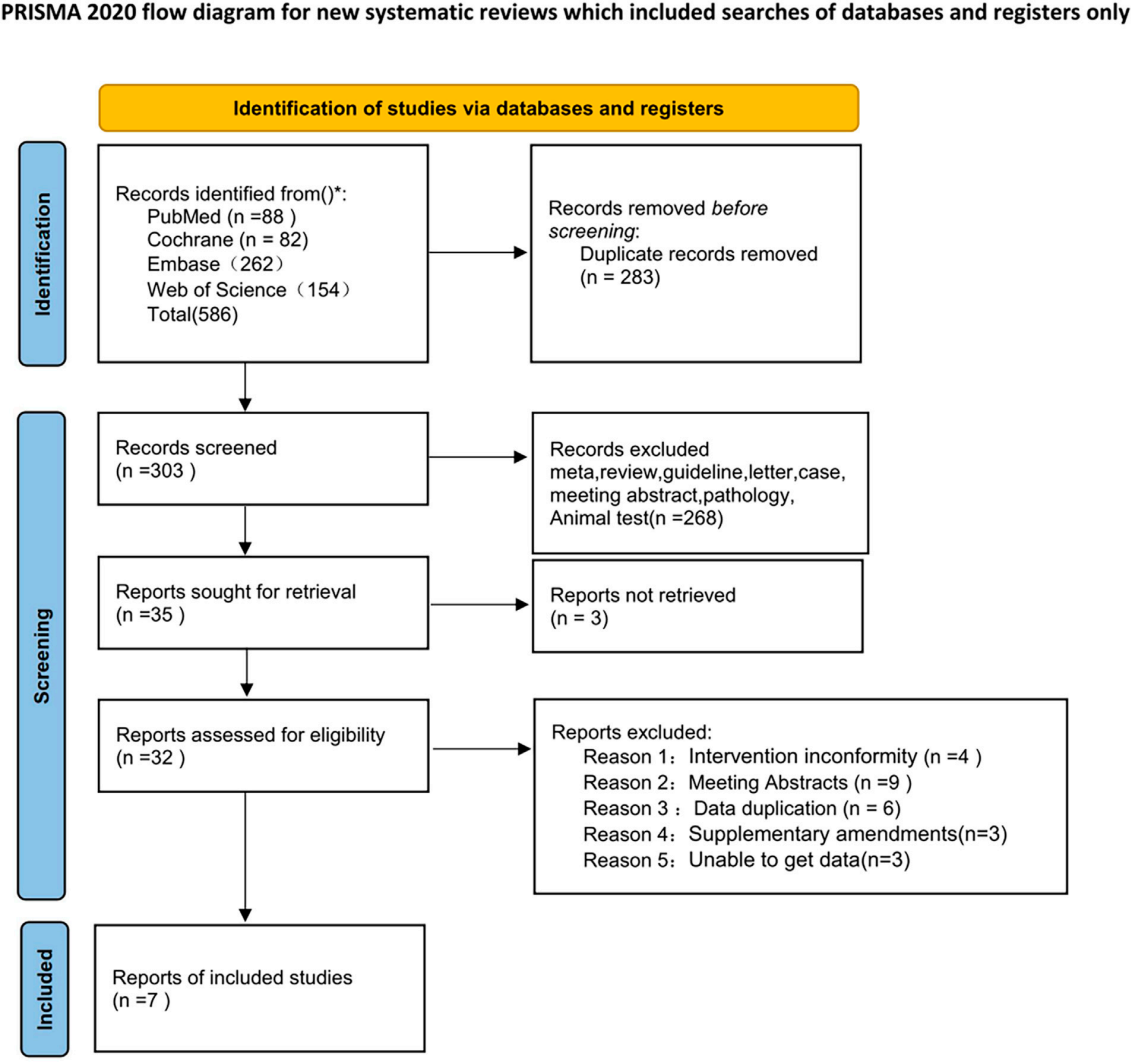
Flow chart.

A total of 586 relevant articles were obtained in the preliminary examination, including 88 in PubMed, 82 in The Cochrane Library, 154 in Web of Science and 262 in EMbase. After layer-by-layer screening, 7 articles (Gooderham et al., 2019; Silverberg et al., 2020; Simpson et al., 2020; Bieber et al., 2021; Simpson et al., 2021b; Eichenfield et al., 2021; Reich et al., 2022) were finally included, among which one RCT was divided into two papers with different research directions, totaling 6 international multi-center RCTs. The specific process and results of literature screening are shown in Figure 1.

### 3.2 Basic characteristics of the study

All the studies included in this work were international multi-center RCTs. Except that the control group carried out by Reich et al. (2022) was Dupilumab, other tests included the placebo control group, and T. Bieber (Bieber et al., 2021) also included the Dupilumab group. The dosage of abrocitinib in most studies were 100 mg or 200mg, which was taken orally once a day for 12 weeks. The course of treatment in individual trials was 16 weeks and 26 weeks. Among the 6 RCTs, 3 subjects included adolescents. In the 6 RCTs, oral antihistamines and emollients without drug effect were allowed. Most of the RCTs prohibited the external use of low and medium acting steroids and calcineurin inhibitors, but two of them (Eichenfield et al., 2021; Reich et al., 2022) were allowed. The basic characteristics of each study are shown in Table 1.

**TABLE 1:** Basic information of included research.

First author	Publication years	Country	Type of literature	Sample size e/C	Experimental group	Control group	Inclusion Criteria	Diagnostic Criteria	Outcome indicator	Age (years old)
T. Bieber	2021	International Multi-center	RCT	464/373	200 mg or 100 mg of abrocitinib orally once daily for 16 weeks	300 mg of dupilumab subcutaneously every other week (after a loading dose of 600 mg), or placebo for 16 weeks	Disease course>1 year	Hanifin and Rajka criteria	①②③⑧	Over 18
							IGA≥3			
							EASI≥16			
							BSA≥10%			
							PP-NRS≥4			
Kristian Reich	2022	International Multi-center	RCT	362/365	200 mg of abrocitinib orally once daily for 26 weeks	300 mg of dupilumab subcutaneously every other week (after a loading dose of 600 mg)	Disease course>6 months	Hanifin and Rajka criteria	①②③⑧	Over 18
							IGA≥3			
							EASI≥16			
							BSA≥10%			
							PP-NRS≥4			
Jonathan I. Silverberg	2020	International Multi-center	RCT	278/52	200 mg or 100 mg of abrocitinib orally once daily for 12 weeks	placebo for 12 weeks	Disease course>1 year	Hanifin and Rajka criteria	①②③④⑤⑥⑦⑧	Over 12
							IGA≥3			
							EASI≥16			
							BSA≥10%			
							PP-NRS≥4			
Lawrence F. Eichenfield	2021	International Multi-center	RCT	183/90	200 mg or 100 mg of abrocitinib orally once daily for 12 weeks	placebo for 12 weeks	Disease course>6 months	Hanifin and Rajka	①②③④⑤⑦	12-17
							IGA≥3			
							EASI≥16			
							BSA≥10%			
							PP-NRS≥4			
Melinda J. Gooderham	2019	International Multi-center	RCT	75/28	200 mg or 100 mg of abrocitinib orally once daily for 12 weeks	placebo for 12 weeks	Disease course>1 year	Hanifin and Rajka	①②③	18-75
							IGA≥3			
							EASI≥12			
							BSA≥10%			
Eric L Simpson	2020	International Multi-center	RCT	272/61	200 mg or 100 mg of abrocitinib orally once daily for 12 weeks	placebo for 12 weeks	Disease cours>1 year	Hanifin and Rajka	①②③④⑤⑥⑦	Over 12
							IGA≥3			
							EASI≥16			
							BSA≥10%			
							PP-NRS≥4			
Eric L. Simpson	2021	International Multi-center	RCT	464/373	200 mg or 100 mg of abrocitinib orally once daily for 12 weeks	placebo for 12 weeks	Disease course>1 year	Hanifin and Rajka	④⑤⑥⑧	18-75
							IGA≥3			
							EASI≥12			
							BSA≥10%			

Note: Outcome indicators ① IGA (Investigator’s Global Assessment); ② EASI (Eczema area and severity index); ③ PP-NRS (Peak Pruritus Numerical Rating Scale); ④POEM(Patient-Oriented Eczema Measure); ⑤PSAAD(Pruritus and Symptoms Assessment for Atopic Dermatitis); ⑥ DLQI (Dermatology Life Quality Index); ⑦CDLQI (Children’s Dermatology Life Quality Index); ⑧HADS(Hospital Anxiety and Depression Scale).

### 3.3 Bias risk assessment results included in the study

**FIGURE 2:**
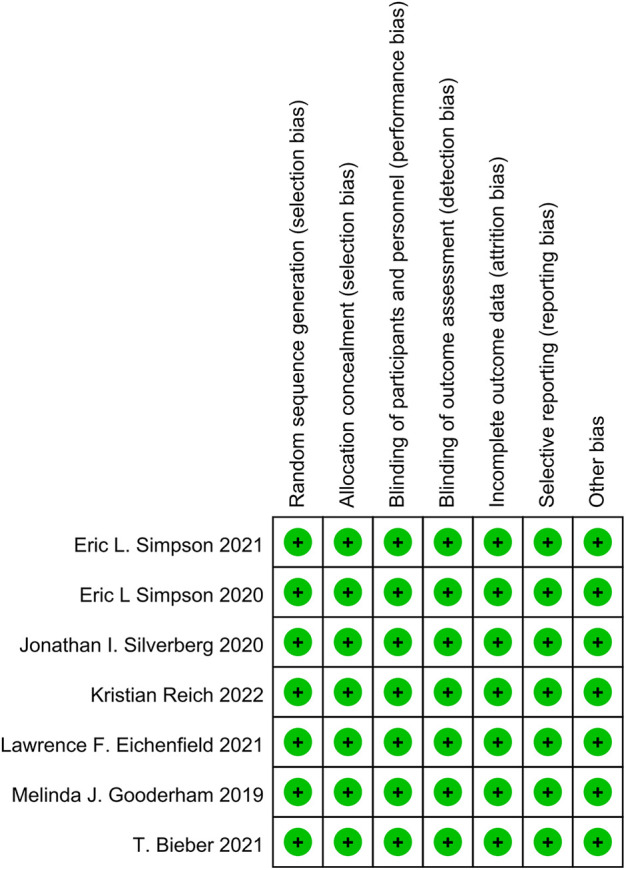
Bias risk assessment chart.

All studies included in this work had clear random methods, allocation concealment, implementation of blind methods and a complete introduction of outcome indicators and were assessed as having a low risk of bias. See Figure 2 for detailed bias risk assessment.

### 3.4 Meta-analysis results

#### 3.4.1 Investigator’s Global Assessment (IGA) meta-analysis results

**FIGURE 3:**
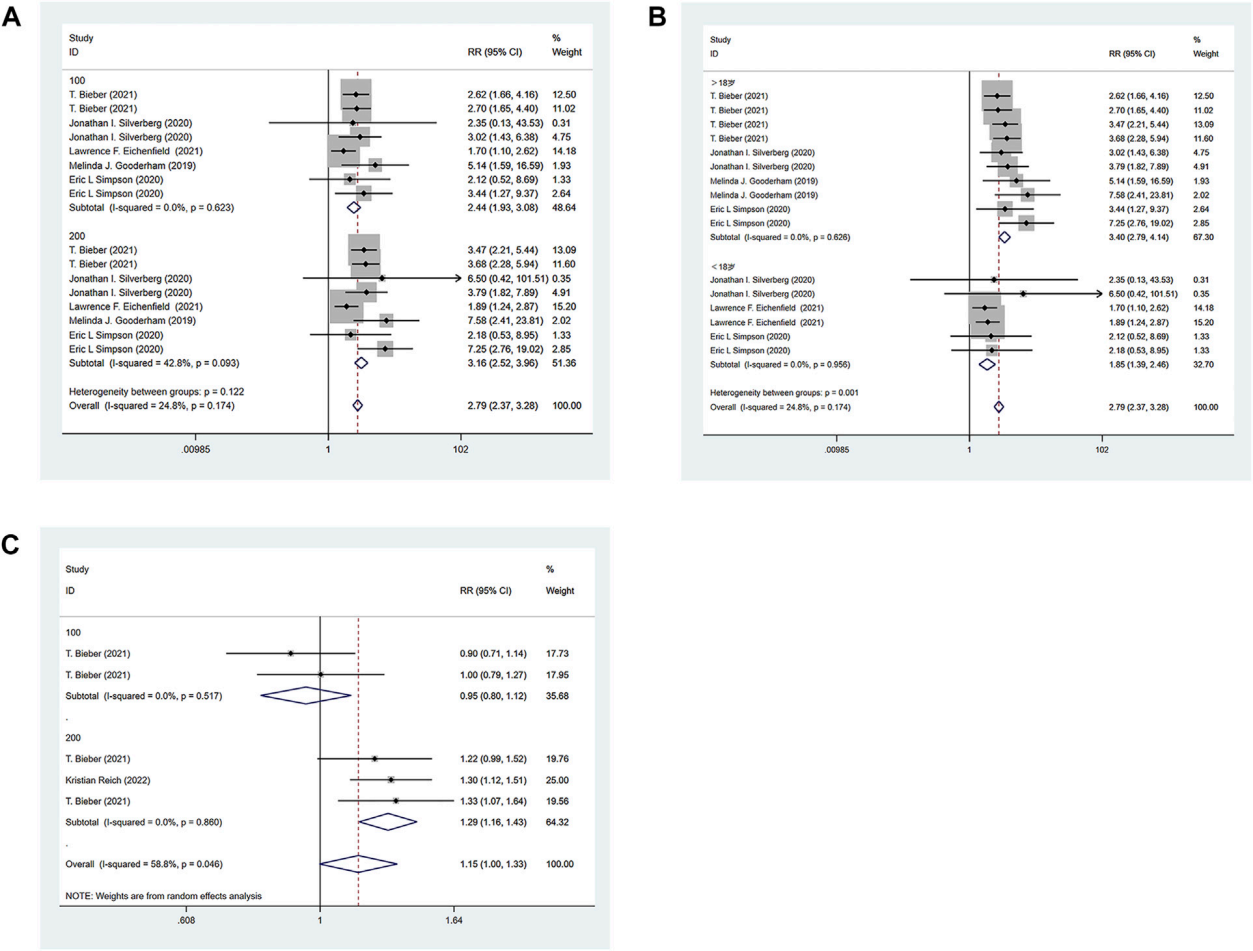
Meta-analysis forest map of IGA. **(A)** Meta-analysis forest map of IGA by dose group; **(B)** Meta-analysis forest map of IGA by age group; **(C)** Meta-analysis forest map of IGA compared with dupilumab.

All the 6 RCTs reported the IGA of the experimental group and the control group. The experimental group was treated with abrocitinib 100 mg and 200mg, while the control group was treated with either placebo or dupilumab 300 mg. Heterogeneity test results showed that (I^2^ = 24.8%) heterogeneity was small, so fixed effect model was adopted for analysis. The results showed that the IGA response of the experimental group was significantly higher than that of the placebo group [RR = 2.79, 95% CI (2.37–3.28)]. Due to differences in doses and ages in various studies, the following IGA response analysis was divided into 100 mg group and 200 mg group for subgroup analysis according to different doses of abrocitinib. The results showed that compared with the placebo group, the effect of abrocitinib 100 mg [RR = 2.44, 95% CI (1.93–3.08)] and 200 mg [RR = 3.16, 95% CI (2.52–3.96)] was significantly better than that of the placebo group. They were also divided into adolescent group and adult group by age. The results showed that compared with the placebo group, the effect of abrocitinib in the adolescent group [RR = 1.85, 95% CI (1.39–2.46)] and the adult group [RR = 3.40, 95% CI (2.79–4.14)] was better than the placebo group. When compared with dupilumab, the heterogeneity test results showed a large (I^2^ = 58.8%) heterogeneity, and the random effect model was therefore adopted for analysis. Compared with dupilumab, there was no statistical difference in IGA response when abrocitinib was administered 100 mg [RR = 0.95, 95% CI (0.80–1.12)]. However, when abrocitinib was administered 200 mg [RR = 1.29, 95% CI (1.16–1.43)], IGA response was higher than that in the control group (Show in Figure 3).

#### 3.4.2 Eczema area and severity index (EASI) meta-analysis results

**FIGURE 4:**
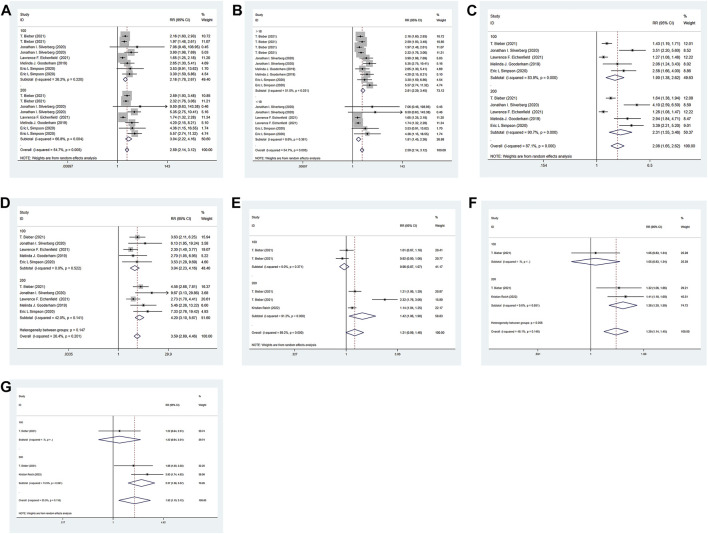
Meta-analysis forest map of EASI-75. **(A)** Meta-analysis forest map of EASI-75 by dose group; **(B)** Meta-analysis forest map of EASI-75 by age group; **(C)** Meta-analysis forest map of EASI-50 by dose group; **(D)**. Meta-analysis forest map of EASI-90 by dose group; **(E)** Meta-analysis forest map of EASI-75 compared with dupilumab; **(F)** Meta-analysis forest map of EASI-90 compared with dupilumab; **(G)** Meta-analysis forest map of EASI-100 compared with dupilumab.

Through the integration comparison with the baseline, improvement greater than 75% is defined as EASI-75, and similarly EASI-50,90,100. The six RCTs included all reported the EASI of the experimental group and the control group. The experimental group was treated with abrocitinib 100 mg and 200mg, while the control group was treated with either placebo or dupilumab 300 mg. The heterogeneity test results showed that (I^2^ = 54.7%) the heterogeneity was large, so the random effect model was adopted for analysis. The results showed that the proportion of patients achieving EASI-75 response in the experimental group 100 mg [RR = 2.18, 95%CI (1.78–2.67)] and 200 mg [RR = 3.04, 95%CI (2.22–4.16)] was higher than that in the placebo group. They were also divided into adolescent group and adult group by age. Adolescent group [RR = 1.81, 95% CI (1.45–2.26)] and the adult group (RR = 2.81, 95% CI (2.29–3.45)] respondents were higher than in the placebo group. For EASI-50 and EASI-90, the experimental 100 mg and 200 mg groups were higher than the control group. Compared with dupilumab, there was no significant difference in EASI-75, EASI-90 and EASI-100 when abrocitinib was administered 100 mg. However, when administered 200 mg, EASI-75 [RR = 1.42, 95% CI (1.06–1.90)], EASI-90 [RR = 1.38, 95% CI (1.20–1.59)], EASI-100 [RR = 2.37, 95% CI (1.58–3.57)] were higher than those in the control group (Show in Figure 4).

#### 3.4.3 Peak Pruritus Numerical Rating Scale (PP-NRS) meta-analysis results

**FIGURE 5:**
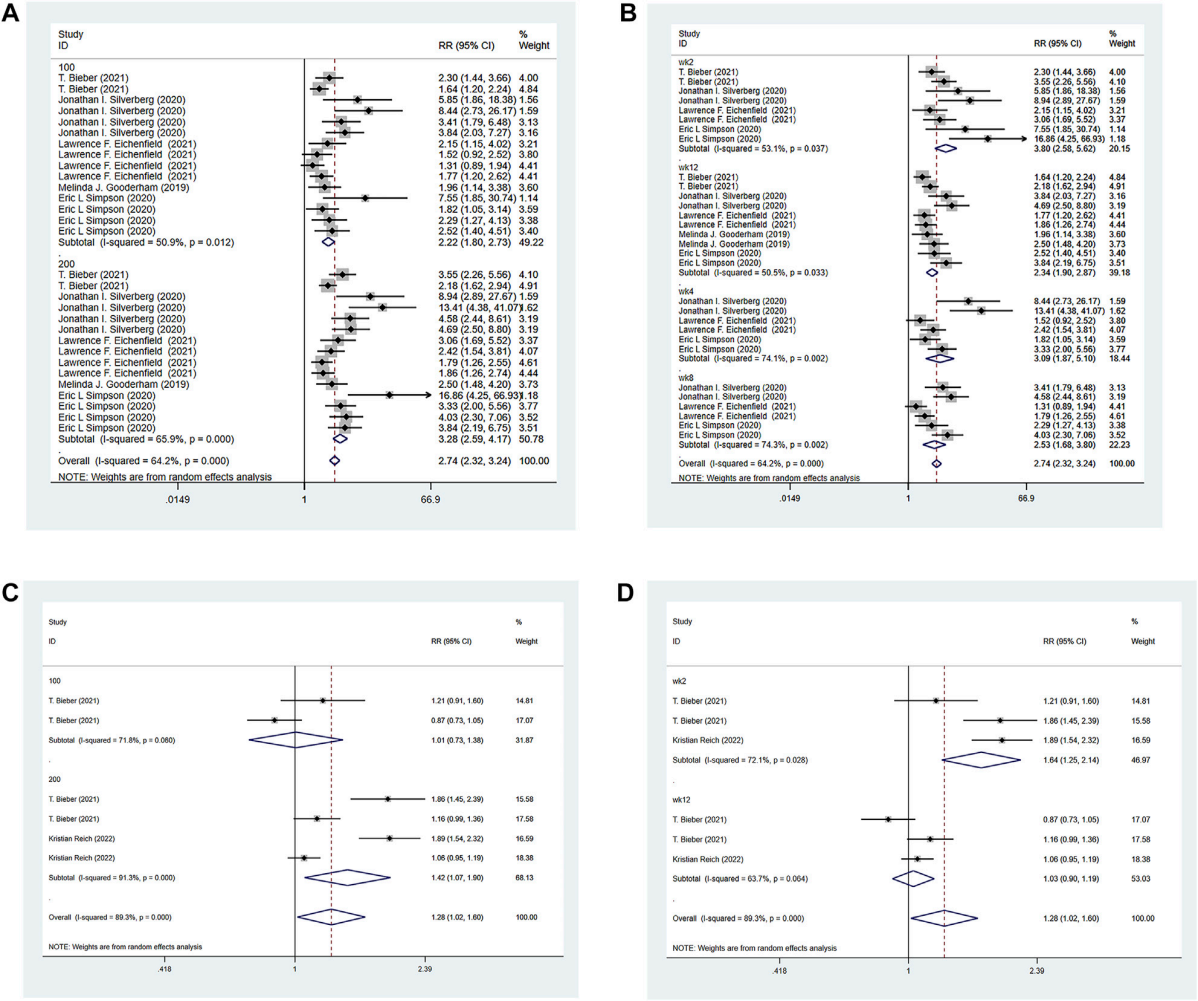
Meta-analysis forest map of PP-NRS4. **(A)** Meta-analysis forest map of PP-NRS4 by dose group; **(B)** Meta-analysis forest map of PP-NRS4 by follow-up time group; **(C)** Meta-analysis forest map of PP-NRS4 compared with dupilumab; **(D)** Meta-analysis forest map of PP-NRS4 compared with dupilumab by follow-up time group.

The six RCTs included all reported PP-NRS4 in the experimental group and the control group. The experimental group was treated with abrocitinib 100 mg and 200 mg, while the control group was treated with either placebo or dupilumab 300 mg. The heterogeneity test results showed that (I^2^ = 64.2%) the heterogeneity was large, so the random effect model was adopted for analysis. The results showed that the PP-NRS4 respondents [RR = 2.74, 95% CI (2.32–3.24)] in the experimental group was higher than that in the placebo group. PP-NRS4 respondents in 100 mg group [RR = 2.22, 95% CI (1.80–2.72)] and 200 mg group [RR = 3.28, 95% CI (2.59–4.17)] were significantly higher than those in placebo group. The subgroup analysis was performed according to the follow-up time. At 2 weeks [RR = 3.80, 95% CI (2.58–5.62)], 4 weeks [RR = 3.09, 95% CI (1.87–5.10)], 8 weeks [RR = 2.53, 95% CI (1.68–3.80)], and 12 weeks [RR = 2.34, 95% CI (1.90–2.87)], the experimental group was higher than the placebo group. Compared with Dupilumab, there was no statistical difference in PP-NRS4 when abrocitinib was administered 100 mg, but PP-NRS4 [RR = 1.42, 95% CI (1.02–1.60)] in the experimental group was higher than that in the control group when abrocitinib was administered 200 mg. By time grouping, at 2 weeks, [RR = 1.64, 95% CI (1.25–2.14)] in the test group was higher than that in the control group, but at 12 weeks, there was no statistical difference between the two groups (Show in Figure 5).

#### 3.4.4 Pruritus and Symptoms Assessment for Atopic Dermatitis (PSAAD) meta-analysis results

**FIGURE 6:**
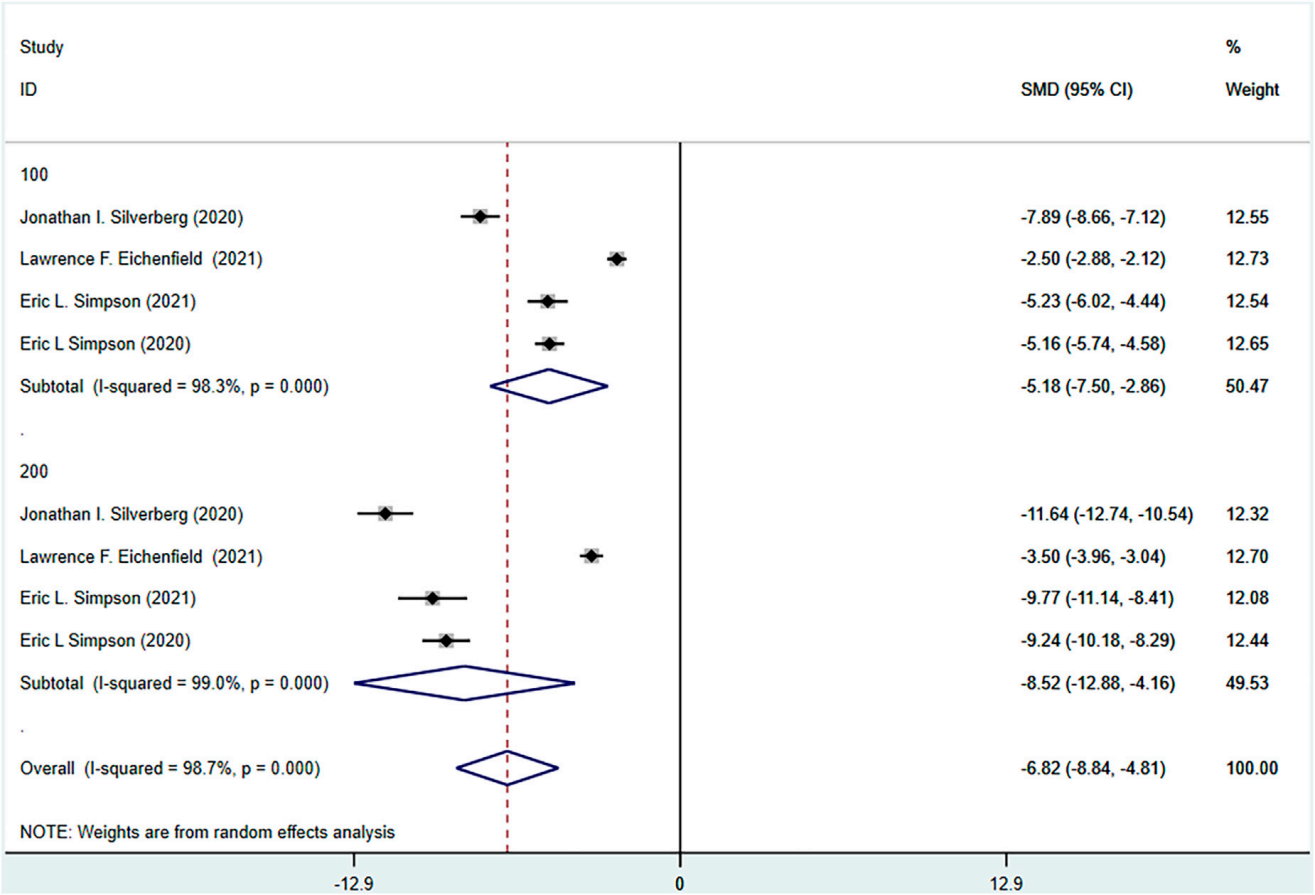
Meta-analysis forest map of PSAAD.

PSAAD of the experimental group and the control group were reported in the four RCTs included. The experimental group was treated with abrocitinib 100 mg and 200 mg, while the control group was treated with placebo. The heterogeneity test results showed that (I^2^ = 98.7%) the heterogeneity was larger than 50%, so the random effect model was adopted for analysis. The results showed that the improvement of PSAAD in the experimental group was better than that in the control group [SMD = −6.82, 95% CI (−8.84∼−4.81)]. In dose grouping, the improvement of PSAAD in 100 mg group [SMD = −5.18,95% CI (−7.50∼−2.86)] and 200 mg group [SMD = −8.52, 95% CI (−12.88∼−4.16)] was better than that in control group (Show in Figure 6).

#### 3.4.5 Patient-oriented eczema measure (POEM) meta-analysis results

**FIGURE 7:**
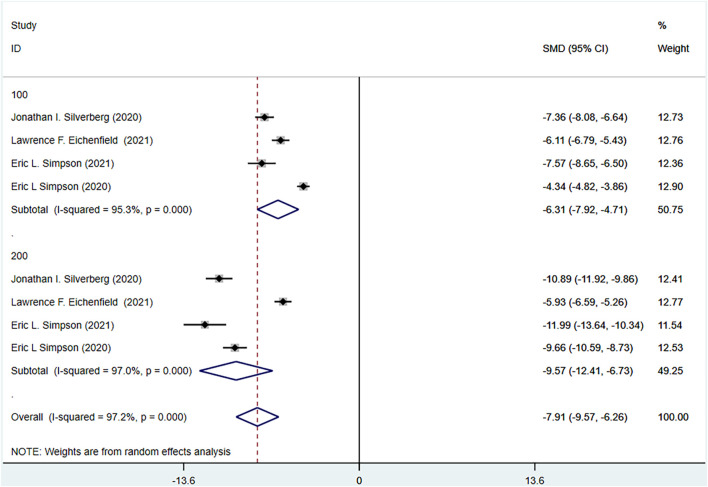
Meta-analysis forest map of POEM.

The POEM of the experimental group and the control group were reported in four RCTs included. The experimental group was treated with abrocitinib100 mg and 200 mg, while the control group was treated with placebo. Heterogeneity results (I^2^ = 97.2%) were greater than 50%, so random effect model was adopted for analysis. The results showed that the improvement of POEM in the experimental group was better than that in the control group [SMD = −7.91, 95% CI (−9.57∼−6.26)]. Grouped by dose, the improvement of POEM in 100 mg group [SMD = −6.31, 95% CI (−7.92∼−4.71)] and 200 mg group [SMD = −9.57, 95% CI (−12.41∼−6.73)] was better than that in control group (Show in Figure 7).

#### 3.4.6 Dermatology life quality index (DLQI) meta-analysis results

**FIGURE 8:**
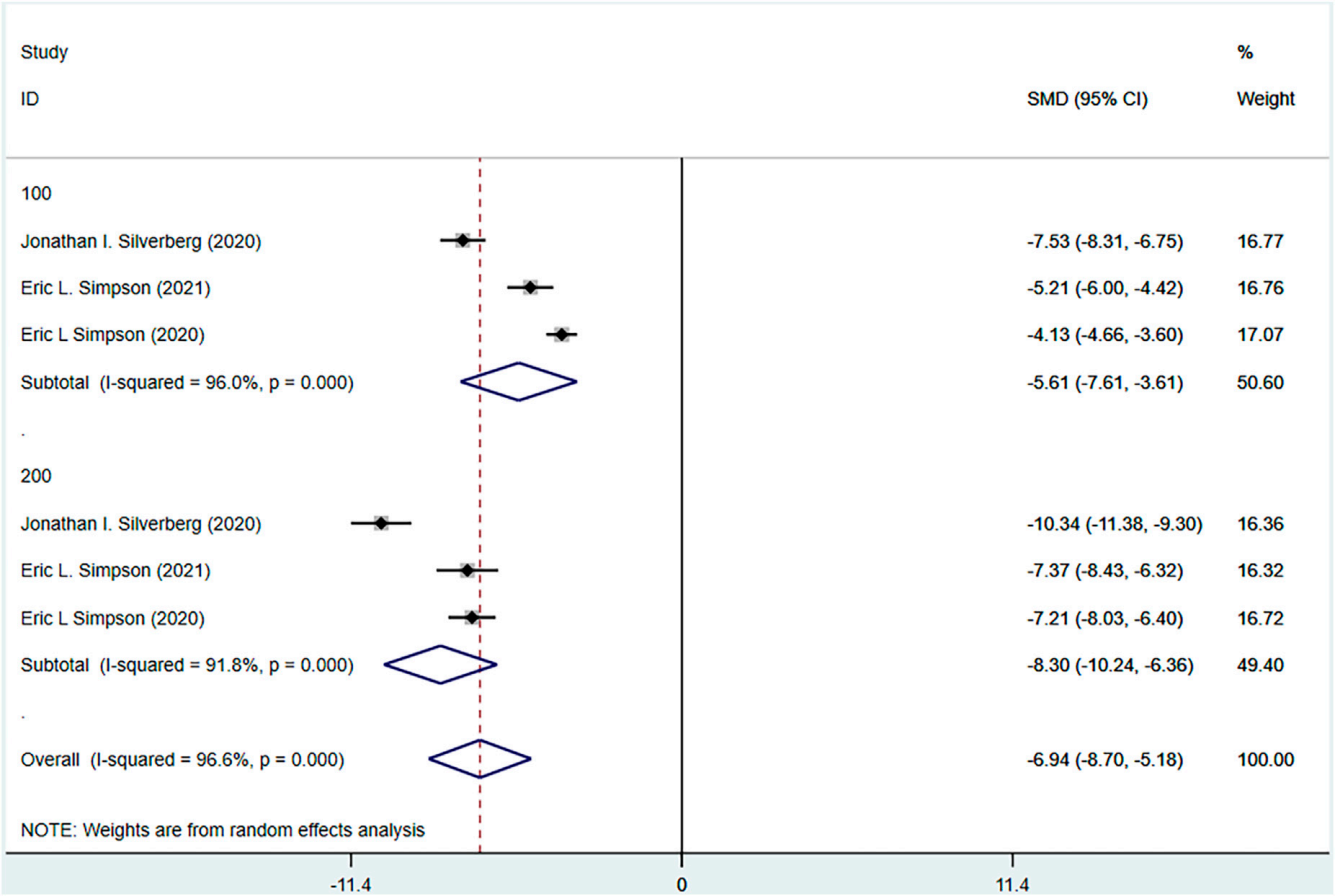
Meta-analysis forest map of DLQI.

The DLQI of the experimental group and the control group were reported in three RCTs included. The experimental group was treated with abrocitinib 100 mg and 200 mg, while the control group was treated with placebo. Heterogeneity results (I^2^ = 96.6%) were greater than 50%, so random effect model was adopted for analysis. The results showed that the improvement of DLQI in the test group was better than that in the control group [SMD = −6.94, 95% CI (−8.70∼−5.18)]. By dose grouping, the improvement of DLQI in 100 mg group [SMD = −5.61, 95% CI (−7.61∼−3.61)] and 200 mg group [SMD = −8.30, 95% CI (−10.24∼−6.36)] was better than that in control group (Show in Figure 8).

#### 3.4.7 Children’s dermatology life quality index (CDLQI) meta-analysis results

**FIGURE 9:**
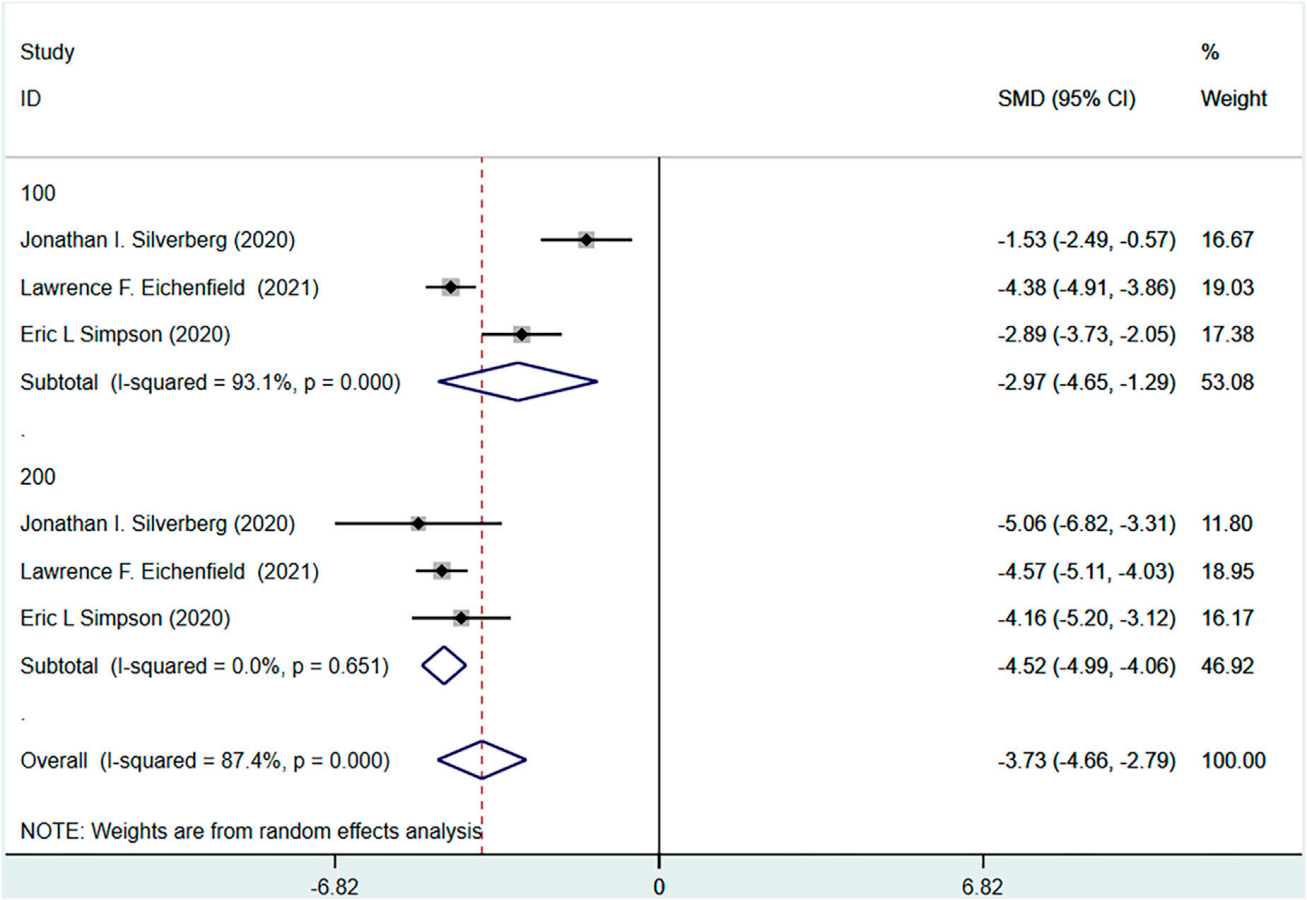
Meta-analysis forest map of CDLQI.

The CDLQI of the experimental group and the control group were reported in the three RCTs included. The experimental group was treated with abrocitinib 100 mg and 200 mg, while the control group was treated with placebo. The heterogeneity result (I^2^ = 87.4%) was greater than 50%, so the random effect model was adopted for analysis. The results showed that the improvement of CDLQI in the experimental group was better than that in the control group [SMD = −3.73, 95% CI (−4.66∼−2.79)]. Grouped by dose, the improvement of CDLQI in 100 mg group [SMD = −2.97, 95% CI (−4.65∼−1.29)] and 200 mg group [SMD = −4.52, 95% CI (−4.99∼−4.06)] was better than that in control group (Show in Figure 9).

#### 3.4.8 Hospital anxiety and depression scale (HADS) meta-analysis results

**FIGURE 10:**
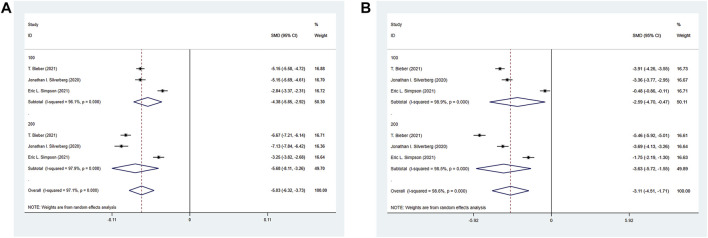
Meta-analysis forest map of HADS. **(A)** Meta-analysis forest map of HADS-Depression by dose group; **(B)** Meta-analysis forest map of HADS-Anxiety by dose group.

The three RCTs included reported the HADS of the experimental group and the control group. The experimental group was treated with abrocitinib 100 mg and 200 mg, while the control group was treated with placebo. As for Depression, the heterogeneity result (I^2^ = 97.1%) was greater than 50%, so the random effect model was adopted for analysis. The results showed that the improvement of depression in 100 mg group [SMD = −4.38, 95% CI (−5.85∼−2.92)] and 200 mg group [SMD = −5.68, 95% CI (−8.11∼−3.26)] was better than that in control group. As for Anxiety, the results showed that the improvement of 100 mg group [SMD = −2.59, 95% CI (−4.70∼−0.47)] and 200 mg group [SMD = -3.63, 95% CI (−5.72∼−1.55)] was better than that of the control group (Show in Figure 10).

#### 3.4.9 Adverse events

The six RCTs included all reported adverse events (AEs) in the experimental group and the control group. The experimental group was treated with abrocitinib 100 mg and 200 mg, while the control group was treated with either placebo or dupilumab. Compared with placebo, there was no statistical difference in the total adverse reactions when 100 mg was administered. When 200 mg was administered, it was slightly higher than the control group [RR = 1.23, 95% CI (1.11–1.37)], but there was no statistical difference in serious adverse reactions between the two groups. The incidence of nausea in 100 mg group [RR = 3.13, 95%CI (1.47–6.69)] and 200 mg group [RR = 7.81, 95%CI (3.84–15.87)] were significantly higher than that in control group. Compared with Dupilumab, there was no statistical difference in the total adverse reactions after administration of 100 mg. When administration of 200 mg, it was slightly higher than the control group [RR = 1.14, 95% CI (1.04–1.24)], but for serious adverse reactions, there was no statistical difference between the two groups. The incidence of nausea in 100 mg [RR = 1.45, 95% CI (0.56–3.75)], 200 mg group [RR = 5.95, 95% CI (2.63–13.48)] were higher than the control group. For conjunctivitis, the incidence in 100 mg [RR = 0.14, 95% CI (0.03–0.59)] and 200 mg [RR = 0.25, 95% CI (0.14–0.45)] were lower than Dupilumab, show in Table 2.

**TABLE 2:** Adverse reaction results.

Study	Side effect	Dosage (mg)	RR	95% CI
Abrocitinib vs. Placebo	adverse events	100	1.09	0.97–1.22
		200	1.23	1.11–1.37
	serious TEAEs	100	0.88	0.42–1.82
		200	0.61	0.27–1.37
	Common side effects			
	Nausea	100	3.13	1.47–6.69
		200	7.81	3.84–15.87
	Nasopharyngitis	100	1.37	0.93–2.03
		200	1.04	0.69–1.57
	Upper respiratory tract infection	100	1.28	0.86–1.90
		200	1.07	0.71–1.62
	Headache	100	1.32	0.75–2.32
		200	1.76	1.03–3.00
	Acne or Folliculitis	100	2.60	0.87–7.75
		200	4.34	1.61–11.71
	Atopic dermatitis	100	0.75	0.49–1.14
		200	0.40	0.24–0.68
Abrocitinib vs. dupilumab	adverse events	100	1.02	0.85–1.21
		200	1.14	1.04–1.24
	serious TEAEs	100	3.05	0.62–14.96
		200	1.02	0.39–2.71
	Common side effects			
	Nausea	100	1.45	0.56–3.75
		200	5.95	2.63–13.48
	Headache	100	0.78	0.35–1.75
		200	1.70	1.10–2.61
	Acne or Folliculitis	100	2.37	0.62–9.07
		200	4.59	2.60–8.09
	Conjunctivitis	100	0.14	0.03–0.59
		200	0.25	0.14–0.45

### 3.5 Sensitivity analysis and publication bias

There were no sensitive issues in the included study. In the Egger’s test of publication bias, *p* > 0.05 was considered as no publication bias. The included indicators EASI-50, EASI-75, EASI-90, PP-NRS4 and Headache had publication bias, while the other indicators were unbiased. The Egger’s test results are shown in Table 3. We used the funnel chart to intuitively display the publication bias, and used Egger’s test to analyze the funnel chart. The analysis results showed that Egger’s test showed that the bias of EASI-50, EASI-75, EASI-90, PP-NRS4, and Headache was (*p* = 0.000, *p* = 0.000, *p* = 0.027, *p* = 0.000, *p* = 0.009). Therefore, considering the publication bias among studies, the funnel chart was further analyzed by using the Trim-and-fill method. After adding 5,7,3,9,4 studies to each indicator model, the funnel chart was symmetrical. At this time, the combined effect amounts were 5.217 (0.902,1.987), 7.293 (1.637,2.840), 32.339 (3.168,3.576), 5.912 (1.568,3.124), and 7.231 (0.925,1.613) respectively. The results of the Trim-and-fill method are shown in Table 4.

**TABLE 3:** Egger’s test results.

Outcome indicator	Item	Effect value	Standard error	95%CI	t-value	*p*-value
IGA	slope	1.810	0.658	0.399–3.222	2.75	0.016
	bias	4.036	1.982	−0.215–8.286	2.04	0.061
EASI-50	slope	0.434	0.235	−0.107–0.945	1.85	0.102
	bias	11.382	1.927	6.939–15.824	5.91	0.000
EASI-75	slope	1.179	0.310	0.513–1.845	3.80	0.002
	bias	6.607	1.427	3.546–9.668	4.63	0.000
EASI-90	slope	0.426	1.355	−2.699–3.551	0.31	0.761
	bias	10.528	3.880	1.581–19.475	2.71	0.027
PP-NRS4	slope	−1.288	0.564	−2.443∼-0.133	-2.28	0.030
	bias	16.147	2.149	11.744–20.550	7.51	0.000
Headache	slope	−1.442	0.957	−3.648–0.764	-1.51	0.170
	bias	5.242	1.530	1.714–8.770	3.43	0.009
Nausea	slope	2.027	6.532	−13.037–17.090	0.31	0.764
	bias	4.907	7.815	−13.113–22.928	0.63	0.548
Upper respiratory tract infection	slope	1.060	0.521	−0.140–2.261	2.04	0.076
	bias	0.308	1.130	−2.298–2.913	0.27	0.792
serious TEAEs	slope	0.501	0.695	−1.101–2.104	0.72	0.491
	bias	0.454	0.795	−1.378–2.287	0.57	0.583

**TABLE 4:** Trim-and-Fill.

outcome indicator	Method	Pooled Est	95%CI	z-value	*p*-value	No. Of studies added
EASI-50	Random	1.444	0.902–1.987	5.217	0.000	5
EASI-75	Random	2.239	1.637–2.840	7.293	0.000	7
EASI-90	Fixed	3.372	3.168–3.576	32.339	0.000	3
PP-NRS4	Random	2.346	1.568–3.124	5.912	0.000	9
Headache	Fixed	1.269	0.925–1.613	7.231	0.000	4

## 4 Discussion

Pruritus is a prominent subjective symptom of severe AD, which can be triggered by excessive cold and overheating stimulation, sweating, emotional changes, and contact with woolens (Silverberg et al., 2018). Our research results based on six randomized controlled trials showed that abrocitinib can rapidly improve the itching symptoms of AD patients. After 2 weeks of treatment, PP-NRS4 values in the experimental group was significantly higher than that in the placebo group, and the effect persisted until the end of treatment. In the second week, the efficacy was comparable to that of Dupilumab when the dosage was 100 mg, and superior to Dupilumab when the dosage was 200 mg. The ISAAC study showed that the prevalence of the same ethnic group varies greatly in different parts of each country or between different countries, suggesting that environmental factors may play an important role. Although the itch mechanism of AD is complex and has not been fully understood, recent evidence shows that histamine is related to interleukin (IL) - 31 (Dillon et al., 2004), IL-13, and IL-4 (Zheng et al., 2009). Moderate-to-severe AD is characterized by an allergic response driven by a subset of immune cells called type 2 helper T-cell (Th2 cells). IL-31, A cytokine released by Th2 cells, is involved in AD-related pruritis through interacting with the neuron-expressed IL-31 receptor A, and plays a role in AD skin inflammation and AD skin barrier destruction. IL-31 binds to the IL-31RA/OSMRβ complex, consists of IL-31 receptor A (IL-31RA) and oncostatin M receptor β (OSMRβ), resulting in subsequent activation of downstream typical kinase pathways, including ERK1/2 MAP kinase, PI3K/AKT, and Janus kinase (JAK) 1/2 signaling pathways (Zhang et al., 2008). All three interleukin cytokines can mediate chronic pruritus via JAK 1/2 signaling pathways (Bonnekoh et al., 2022). Abrocitinib is an oral selective JAK-1 inhibitor that improves pruritus by directly inhibiting neuronal JAK1 by inhibiting the JAK1 pathway.

IGA is an important indicator for clinical investigators to evaluate AD patients (Simpson et al., 2022). EASI assesses the extent of disease in AD by considering eczema, induration, excoriation, and lichenification (Rehal and Armstrong, 2011). PSAAD contains 11 relevant symptoms in patients with AD (itch, dryness, redness, flaking, discolouration, pain, bleeding, cracking, bumps, swelling, and weeping/oozing) (Hall et al., 2021). POEM is recommended by the Harmonising Outcome Measures for Eczema initiative as the core outcome instrument for measuring patient-reported symptoms in AD trials (Grant et al., 2019). The meta results showed that oral abrocitinib could effectively improve the symptoms and signs of patients, and the IGA and EASI-75 respondents were significantly higher than those in the placebo group, and these results showed superiority in both adults and adolescents. EASI-50 and EASI-90 respondents were significantly higher than the control group. PSAAD and POEM scores decreased more significantly than those at baseline. Compared with dupilumab, when administered with abrocitinib 100 mg, the efficacy of both drugs was equivalent, but when administered with 200 mg, abrocitinib was superior to dupilumab, and IGA, EASI-75, EASI-90 and EASI-100 showed statistical differences with abrocitinib 200 mg. Other studies (Alexis et al., 2022; Gooderham et al., 2022; Shi et al., 2022) showed that for those patients who did not respond to the biological agent dupilumab, the EASI-75 rate could reach 80.0% after 12 weeks of treatment with abrocitinib 200 mg (Shi et al., 2022). In addition, abrocitinib showed rapid and sustained improvement in all areas of the body, including difficult-to-treat areas such as the head and neck, and 77.4% (200 mg group) and 51.9% (100 mg group) of abrocitinib maintained EASI-50 after 4 weeks of discontinuation.

The benefits of treatment go beyond improving AD symptoms and have other meaningful effects on the lives of patients. Such as daily activities, personal relationships, symptoms and feelings, leisure activities, and work and school efficiency via DLQI. Quality of life considerations are an important element in assessing treatment response in adolescents with AD, and the Harmonising Outcome Measures for Eczema initiative recommends applying CDLQI monitoring to clinical quality of life in adolescents with AD (Cork et al., 2022). As for mental health, HADS-depression and HADS-anxiety subscale scores reflects its clinical significance. Not only alleviating key signs and symptoms, but also improving downstream mental health comorbidities, it has some improvement in adolescent and adult patient population. Meta results showed that after treatment, DLQI, CDLQI and HADS had a significant downward trend. Its heterogeneity was high, which may be related to the few original studies or the large differences between samples.

As for adverse events, when the dosage was 100 mg or 200 mg, the adverse reactions caused by the drug abrocitinib to the body became more obvious with the increase of the dosage. In the report of adverse events, there was no significant difference between 100 mg and placebo, but when 200 mg was given, the adverse reactions were higher than placebo. However, no matter the dosage of 100 mg or 200 mg, there was no evidence that abrocitinib will cause serious adverse reactions. Most AEs were mild, self-limited, and did not require interruption or permanent discontinuation of abrocitinib therapy. The most common dose-related, drug-related AEs included nausea, headache and acne. These symptoms usually first appeared within 2 weeks of treatment initiation but are rarely severe enough to lead to discontinuation of treatment (Simpson et al., 2021). Acne events were seen across the JAK class, however further research is needed to understand its pathogenesis (Guttman-Yassky et al., 2020). When the dosage was 100 mg, the occurrence of adverse events was equivalent to that of dupilumab, but when the dosage was 200 mg, the incidence of adverse events of abrocitinib was slightly higher. However, when the dosage of abrocitinib was 100 mg or 200 mg, the occurrence of conjunctivitis events was significantly lower than that of dupilumab. In conclusion, the rates of common adverse effects were similar or slightly higher with abrocitinib than with placebo or Dupilumab, although no serious adverse effects occurred while the efficacy of abrocitinib was very significant. Therefore, the adverse reactions and therapeutic effects can be considered according to the specific circumstances. In the future, more samples are needed to prove the safety and efficacy of abrocitinib.

Compared with the previous meta (Meher et al., 2022), the advantage of this study is the inclusion of results from two new high-quality RCTS. The safety and effectiveness of the drug in adolescent patients were confirmed by the subgroup of population age. POEM, PSAAD, DLQI, CDLQI, HADS and other indicators have been added to help us more comprehensively understand the impact of abrocitinib on patients with moderate-to-severe AD, including the impact on symptoms and signs, as well as the quality of life, anxiety, depression and other emotional aspects. By comparing with the biological agent dupilumab, we can more intuitively see the effectiveness of abrocitinib in treating moderate and severe AD patients in adolescents and adults.

However, this study also has some limitations. The treatment time of different studies was slightly different. Most of the studies were 12 weeks, but there were also 16 weeks and 26 weeks. There were two other studies that allowed the external use of moderate and low effect steroids and calcineurin inhibitors. Whether they would interfere with the therapeutic effect needs further study. At the same time, the number of included research literatures was relatively small, and some research results were highly heterogeneous. It is expected that more high-quality researches will be further demonstrated and analyzed in the future.

## 5 Conclusion

In conclusion, this meta-analysis preliminarily proved that abrocitinib is safe and effective for adolescent and adult patients with moderate-to-severe AD who do not respond to conventional therapies and require systematic treatment. It can rapidly relieve itching, effectively improve symptoms and signs, and improve quality of life, and the dosage of 200 mg is better than 100 mg. The good effect of abrocitinib 100 mg was equivalent to that of dupilumab, while the effect of abrocitinib 200 mg was superior to that of dupilumab. However, in future studies with a larger sample size, it is necessary to compare the efficacy and safety of abrocitinib and active drugs to provide clear evidence about the drug.
